# Balance and Walking Speed Outcomes in Individuals Receiving Inpatient Rehabilitation for Acute Cerebellar Stroke

**DOI:** 10.3390/nursrep14040214

**Published:** 2024-10-10

**Authors:** Uzair Hammad, Abigail W. Anderson, Emma Scammon, Reid Whiting, Juan Pablo Rodriguez, Rolando T. Lazaro, Morris Casano Beato

**Affiliations:** 1 Orlando Health Advanced Rehabilitation Institute, Orlando, FL 32806, USA; uzair.hammad@orlandohealth.com; 2 School of Kinesiology and Rehabilitation Sciences, College of Health Professions and Sciences, University of Central Florida, Orlando, FL 32816, USA; 3 Department of Physical Therapy, California State University Sacramento, Sacramento, CA 95819, USA

**Keywords:** cerebellar stroke, acute care, physical therapy, nursing

## Abstract

Background/Objectives: Cerebellar strokes account for only 2–3% of all strokes occurring annually in the United States but represent a disproportionally higher share of morbidity and mortality. Evidence examining the effect of inpatient rehabilitation on functional outcomes following a cerebellar stroke is limited. This study aimed to examine the effects of inpatient rehabilitation on balance and walking speed in individuals with cerebellar stroke. A secondary purpose of this study was to examine the length of inpatient rehabilitation stay of the included patients. Methods: A retrospective analysis was conducted using review of patient records during their inpatient rehabilitation stay from January 2021 to February 2022 at a large hospital system in the southeast United States. Balance and gait outcomes were examined on admission and discharge from inpatient rehabilitation that included physical therapy interventions. A paired *t*-test examined for changes in outcomes from admission to discharge. Pearson correlation examined for the association between length of stay and outcomes. Results: A total of 15 records were reviewed. There were significant improvements in the Berg Balance Scale (BBS), Postural Assessment Scale for Stroke (PASS), and the 10-Meter Walk Test (10MWT) (*p*’s < 0.01) from admission to discharge with large effect sizes (range d = 0.70–1.67) following inpatient rehabilitation. The average length of stay was 12.67 days (SD = 6.5) and the mean total hours of combined occupational, physical, and speech therapy was 27.33 (SD = 6.52) h. There was a moderate association between length of stay and PASS (r = 0.525, *p* = 0.04) and BBS (r = 0.546, *p* = 0.04) outcomes. Conclusions: Patients who underwent inpatient rehabilitation following acute cerebellar strokes demonstrated improvements in balance and gait speed. Study results could assist clinicians designing interventions for patients with cerebellar strokes in the inpatient rehabilitation setting.

## 1. Introduction

Cerebellar strokes are infarcts or hemorrhages of the vessels targeting the cerebellum [1]. Cerebellar strokes account for only 2–3% of all strokes occurring annually in the United States but represent a disproportionally higher share of morbidity and mortality. In a retrospective study of individuals admitted to the hospital with a cerebellar stroke, 17.7% of individuals died due to complications related to the stroke [2]. Poor outcomes were observed for 38.0% of individuals at discharge and for 31.6% of individuals at six months [2]. Complications related to the cerebellar stroke included brainstem compression and development of hydrocephalus [2]. Long-term negative functional outcomes are also impacted by impairments in ambulation and movement coordination that result in a lower quality of life. Given the cerebellum’s role in gait and balance, patients with a cerebellar stroke may present with impairments in these areas. However, movement disorders after a stroke may vary depending on the location of the lesion and type of stroke. Patients with stroke involving the cerebral hemispheres may impact the primary and supplementary motor areas resulting in hemiparesis. Rehabilitation may need to be modified depending on the location and type of stroke.

An inpatient rehabilitation facility (IRF) qualifies for reimbursement under the Center for Medicare and Medicaid Services (CMS) prospective payment system by adhering to specific CMS regulations. Patients must participate in an intensive, multidisciplinary rehabilitation program, attending 3 h of therapy on at least five of seven consecutive days. Alternatively, they may receive 15 h (900 min) of therapy over seven days if they cannot meet the 3 h daily requirement due to factors such as low endurance. The therapies included are physical therapy (PT), occupational therapy (OT), speech and language pathology (SLP), and use of orthotic and prosthetic services. Daily notes and team conference documentation provide evidence of the patient’s functional improvement [3,4].

Given the complex needs of patients with cerebellar stroke, care under an interdisciplinary team in inpatient rehabilitation is often recommended following discharge from the hospital. Rehabilitation as a part of a comprehensive plan of care is recommended in the literature as patients benefit from a multidisciplinary approach that includes interventions focused on balance impairments [1]. While in inpatient rehabilitation, nurses who possess advanced training and experience in rehabilitation care have responsibilities including providing direct patient care, administering medications, and educating patients and their families about stroke recovery and prevention. Patients must be examined by a rehabilitation-trained physician at least three times per week. Additionally, each patient must have a case manager or social worker, and there must be a weekly interdisciplinary team conference led by the physician. Upon admission, there must be an expectation that the patient will benefit from rehabilitation services. Rehabilitation services, including physical, occupational, and speech therapy, have the potential to enhance ambulation and balance outcomes in patients with neurological injuries. 

The literature on inpatient rehabilitation management following a cerebellar stroke is limited. In a retrospective review of 58 patients with cerebellar stroke admitted to inpatient rehabilitation, mean Functional Independence Measure (FIM) scores improved from 65.5 to 89.9 after the rehabilitation stay [5]. However, specific interventions were not described and balance and walking speed outcomes were not assessed. Additional studies have examined the effects of specific interventions, including treadmill training, on outcomes in individuals with cerebellar stroke. Two weeks of treadmill training resulted in full recovery of postural stability three months later in patients with acute and isolated cerebellar stroke (n = 23) [6]. However, treadmill training did not have significant effects on gait ataxia [6]. Intensity of the treadmill speed was the only variable adjusted for during the intervention, and treadmill training was performed in patients who were walking independently. It is therefore unknown whether treadmill training is beneficial for patients who require assistance with ambulation. To quantify improvements in postural stability, the authors also used dynamic posturography, which may be inaccessible in many inpatient settings [6]. Regarding length of stay, the authors found only one retrospective review, which reported an average length of stay of 21.9 days in inpatient rehabilitation following cerebellar infarction without accompanying information regarding what interventions were implemented [6].

The current literature supports postural training and treadmill training to improve functional outcomes and postural stability in people with cerebellar strokes [5,6]. The recent literature supports the novel use of virtual reality in patients with acute cerebellar stroke to improve function such as reaching accuracy [7]. However, a significant gap remains in the reporting of the effects of rehabilitation interventions on balance and gait outcomes in this population. Motor function appears to impact outcomes in patients with cerebellar stroke [8]. In addition, outcomes that have been validated for patients with stroke and are easily accessible to clinicians, such as the Berg Balance Scale (BBS), Postural Assessment Scale for Stroke (PASS), and the 10-Meter Walk Test (10MWT) have not been reported for individuals receiving inpatient rehabilitation for cerebellar stroke [9].

Walking speed has been accepted as a predictor for safe household, limited community, or community ambulation for multiple populations including individuals with stroke and older adults. Established cutoff values for categories of ambulation range from 0.4 m/s to 0.8 m/s, where gait speed greater than 0.4 m/s is considered a household ambulator, greater than 0.6 m/s is considered a limited community ambulator, and greater than 0.8 m/s is considered a community ambulator [10]. Specifically for individuals who have had a stroke, it is found that being able to transition from these categories is also linked to increased functionality and quality of life [11]. In addition to being able to improve from one category of gait classification to another, it has also been shown to be beneficial to improve gait speed by 0.16 m/s for patients in the subacute phase post-stroke [12]. While the literature shows the benefits of improving gait speed within stroke populations, especially in the subacute phase, few studies investigated the specific impact of inpatient rehabilitation on measures of balance and gait speed. 

The current literature supports positive outcomes following inpatient rehabilitation for patients with large-vessel cortical strokes [13]. However, evidence examining the effect of inpatient rehabilitation following cerebellar strokes remains limited. Improved postural control and walking ability resulting from rehabilitation interventions could significantly enhance activity and participation, thereby improving quality of life. Moreover, there are limited data on the length of stay for inpatient rehabilitation in individuals with cerebellar strokes. Given the higher rates of morbidity and complications in these patients, such data could inform the development of inpatient rehabilitation plans of care and discharge planning. Therefore, the primary purpose of this study is to examine the effects of inpatient rehabilitation on balance and walking speed assessed with the Postural Assessment for Stroke Scale (PASS), Berg Balance Scale (BBS), and 10-Meter Walk Test (10MWT) on individuals with cerebellar stroke. A secondary purpose of this study is to report the mean length of stay in inpatient rehabilitation for individuals with this diagnosis and correlate length of stay with balance and walking outcomes. This retrospective study is expected to help guide rehabilitation clinicians in the management of this unique patient population to maximize patient outcomes in inpatient rehabilitation.

## 2. Materials and Methods

### 2.1. Participants

As a retrospective analysis, consecutive medical records of patients diagnosed with cerebellar stroke admitted between January 2021 and February 2022 to inpatient rehabilitation in a large, comprehensive medical facility located in the southeastern United States were reviewed. Participants who met the following eligibility criteria were reviewed. Inclusion criteria for review: (1) cerebrovascular accident lesion that included but was not isolated to the cerebellum; (2) admission to inpatient rehabilitation; and (3) adults 18 years of age and older. Exclusion criteria: (1) history of a previous stroke and (2) presence of another medical condition that would impact the balance and coordination of the individual such as a past or current diagnosis of Parkinson disease, spinal cord injury, or other similar medical conditions that impact balance and motor performance. 

The study was reviewed and approved by the Orlando Health Institutional Review Board (IRB) and acknowledged by the University of Central Florida Institutional Review Board to protect the rights and welfare of human subjects. The principles outlined in the Declaration of Helsinki were followed. Chart reviews were performed on records that satisfied the inclusion criteria. Patient records were de-identified prior to review. Due to the retrospective nature of this study, written informed consent was not required and a waiver of consent was approved by the IRB.

After identifying patients eligible for the retrospective chart review based on inclusion/exclusion criteria, de-identified data were extracted to a computer-based data extraction form. The data collection form was designed to include the following information: participant demographics; length of inpatient rehabilitation stays; admission and discharge scores on the following tests: the Berg Balance Scale, the Postural Assessment Scale for Stroke (PASS), and the 10-Meter Walk Test (10MWT; and physical therapy interventions performed. 

### 2.2. Data Collection Tools

Minutes of therapy received, treatment documentation, outcome measure details, and all other data utilized were diligently recorded using electronic medical record software and were available for use in this study. Outcome measures were administered and completed by the licensed physical therapists employed within the inpatient rehabilitation facility (IRF). The facility implemented monthly in-services to standardize outcome measure assessments to ensure reliable and valid interpretation for 18 months prior to this study. Each physical therapist used the same standardized tools, surfaces, and equipment required for the outcome measures as part of the department policy for improved standardization and interrater reliability. 

Interventions were delivered by licensed physical and occupational therapists and speech-language pathologists. This study intended to synthesize and report the interventions and related outcomes of patients with cerebellar strokes who were admitted for inpatient rehabilitation and received interventions in a non-controlled clinical setting to improve translation and application of these results to clinical practice. While the facility implemented monthly in-services reviewing the literature and evidence-based interventions with staff therapists, intervention choice was ultimately up to the individual clinician’s discretion. 

Physical therapy interventions were designed to achieve the maximum number of steps within a given therapy session, utilizing clinician decision-making to determine the most feasible, efficient, and safe method to maximize stepping practice. Options available to the clinicians at this facility include static/dynamic overhead harness systems which were used for fall prevention only, Arjo walker systems to provide a weightbearing platform for the upper extremities, parallel bars, and typical medical equipment such as canes and walkers. Clinicians primarily focused on a combination of harness supported treadmill training and overground gait training with a goal of achieving 70–85% of the patients age-predicted heart rate maximum (HRMax), consistent with high intensity gait training recommendations [10]. Of note, although clinical documentation from the therapists’ revealed the goal of reaching patients target HR during gait training, clinicians did not consistently identify specific heart rate parameters (time spent in 70–85% HRzone, MaxHR achieved, HR avg, etc.) to determine if patients achieved the dosage goal. 

Review of the patients’ charts also revealed that physical therapists opted to include balance training for higher functioning patients and transfer training for lower functioning patients, in addition to gait training depending upon the patient’s tolerance to the activity. Balance training consisted of static/dynamic standing balance activities and was individualized for patients so they required no more than minimal assistance (25% or less) to maintain standing stability. Balance intervention selections listed from least difficult to mostt difficult are as follows: static standing on firm surface with eyes open, static standing on firm surface with eyes closed, static standing on compliant surface with eyes open, static standing on compliant surface with eyes closed, and maintaining balance during dynamic stepping exercises. Activities were further progressed with decreasing base of support, inclusion of head turns in pitch/yaw planes, and reaching outside base of support for cones.

Occupational therapists prioritized basic Activities of Daily Living (ADL) and Instrumental Activities of Daily Living (iADL) training. Interventions were completed sitting for patients who required more than minimal assistance to sustain standing and standing for patients who required minimal assistance (25% or less) or less during standing ADL task. Activities included dressing, grooming, bathing, eating, cooking, and therapeutic exercises for the upper extremities with a goal of improving fine motor skills, coordination, and cognitive function. Adaptive techniques were also utilized to help patients regain independence in self-care, work, and leisure activities.

Speech-language pathologists emphasized interventions targeted at improving swallowing difficulties (dysphagia) and ensuring safe and effective feeding practices, speech exercises, and cognitive training. Interventions are summarized in Table 1.

### 2.3. Outcome Measures

Measures utilized to assess outcomes in balance and walking speed included the PASS, BBS, and the 10-Meter Walk Test for Self-Selected Speed (10MWT-SS) and Fastest Speed (10MWT-F).

The PASS has been validated in patients with stroke with a cutoff score of 12.5 points for the prediction of independent ambulation upon discharge (Sensitivity 78.9%, Specificity 83.7%) [14]. The BBS has been validated for individuals with stroke with a cutoff score of 46.5 with lower scores indicating increased fall risk (Sensitivity 75%, Specificity 76.9%) [15]. For the 10MWT, gait speed cutoff scores in stroke range between <0.4, 0.4–0.8, and >0.8 m/s, indicating the ability to ambulate household, limited community, and community distances, respectively [10].

### 2.4. Statistical Analysis

SPSS Statistical Software, version 29.0 (IBM Statistics, Armonk, NY, USA) was used for the statistical analysis. The 10MWTSS, 10MWTF, BBS, and PASS were the main outcome measures for the study. All the outcome measures pre- and post-intervention were extracted from the medical records. To examine outcomes after inpatient rehabilitation, a paired *t*-test examined for changes in the PASS, BBS, 10MWTS-SS, and 10MWT-F from admission to discharge. Alpha levels were set to *p* < 0.05. Next, to determine the impact of length of stay and minutes of therapy on discharge outcomes, difference scores in each outcome were calculated with the following formula: discharge score-admission score. Separate Pearson correlations examined for the association between length of stay in days and hours in therapy with the calculated difference for the PASS, BBS, 10MWT-SS, and 10MWT-F. Correlations of 0.2 indicated a weak correlation, 0.5 indicated a moderate correlation, and 0.7 or greater indicated a strong correlation [16].

## 3. Results

### 3.1. Participants

The baseline characteristics of the individuals in this study are presented in Table 2. Fifteen patients were identified that satisfied the inclusion criteria (9 men, 6 women). The patients’ ages ranged from 60 to 81 years old (mean age = 70.26, SD = 6.63). Seven patients had infarction (46.6%) involving only the left cerebellar hemisphere and three patients had infarction (20.0%) involving only the right cerebellar hemisphere. Fourteen patients (93.3%) had isolated cerebellar stroke, while one patient (6.6%) had cerebellar and cerebral infarctions. All patients were in the acute stage of the cerebellar stroke when they underwent inpatient rehabilitation.

A total of 66.7% of the participants were characterized as household ambulators, 33.3% were classified as limited community ambulators, and 0.0% were community ambulators using self-selected walking speed at admission. A total of 20% were classified as household ambulators, 46.6% were limited community ambulators, and 33.3% were classified as community ambulators using self-selected walking speed at discharge.

### 3.2. Effects of Inpatient Rehabilitation

#### 3.2.1. PASS

**Figure 1:**
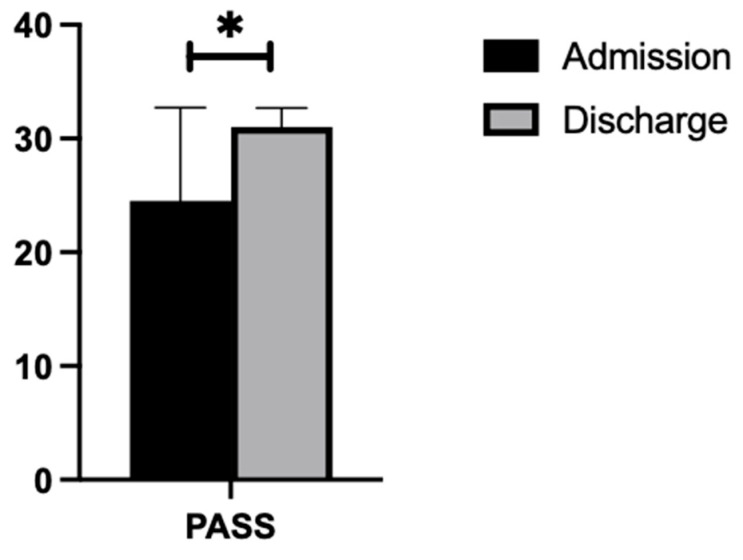
PASS scores at admission and discharge. * significance *p* < 0.05.

As demonstrated in Figure 1, a paired-sample *t*-test indicated that on average, participants with a cerebellar stroke demonstrated a significant increase in PASS scores from admission (M = 24.53, SD = 8.17) to discharge (M = 31.00, SD = 1.69). This difference (−6.47, BCa 95% CI [−11.01, −1.91]) was significant (t(14) = −3.05, *p* = 0.009) and represented a large effect size (d = 0.79).

#### 3.2.2. BBS

**Figure 2:**
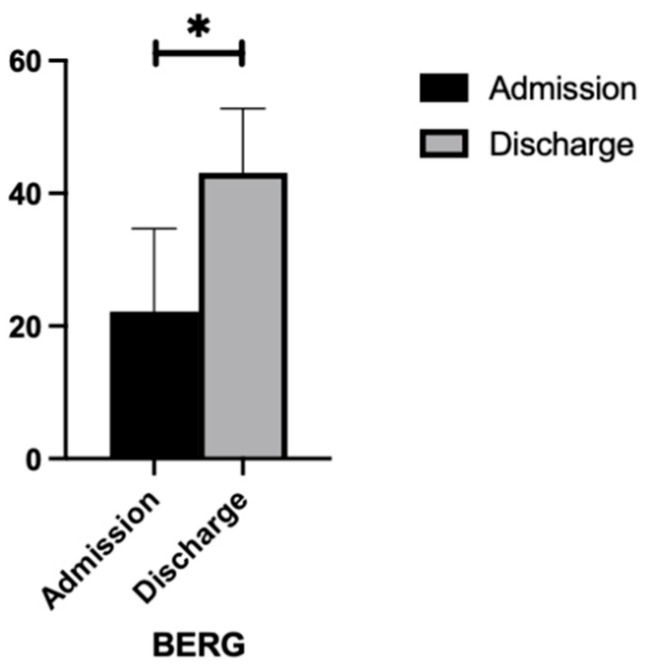
BBS scores at admission and discharge. * significance *p* < 0.05.

As demonstrated in Figure 2, a paired-samples *t*-test indicated that, on average, participants with a cerebellar stroke demonstrated a significant increase in BBS scores from admission (M = 22.20, SD = 12.47) to discharge (M = 43.06, SD = 9.71). This difference (−20.87, BCa 95% CI [−27.76, −13.97]) was significant (t(14) = −6.49, *p* < 0.001) and represented a very large effect size (d = 1.67).

#### 3.2.3. 10MWT-SS

**Figure 3:**
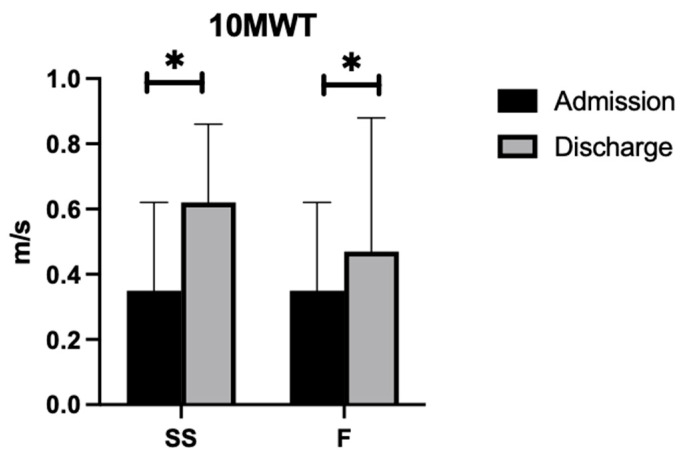
10MWT scores at admission and discharge. * significance *p* < 0.05.

As demonstrated in Figure 3, a paired-samples *t*-test indicated that, on average, participants with a cerebellar stroke demonstrated a significant increase in 10MWT-SS scores from admission (M = 0.3513, SD = 0.27) to discharge (M = 0.6180, SD = 0.24). This difference (−0.27, BCa 95% CI [−0.38, −0.16]) was significant (t(14) = −5.21, *p* < 0.001) and represented a large effect size (d = 1.35).

#### 3.2.4. 10MWT-F

As demonstrated in Figure 3, a paired samples *t*-test indicated that, on average, participants with a cerebellar stroke demonstrated a significant increase in 10MWT-F scores from admission (M = 0.3513, SD = 0.27) to discharge (M = 0.47, SD = 0.41). This difference (−0.40, BCa 95% CI [−0.54, −0.26]) was significant (t(14) = −6.08, *p* < 0.001) and represented a large effect size (d = 1.57).

### 3.3. Length of Stay and Minutes of Therapy

The average length of stay was 12.67 days (SD = 6.5) and the mean total hours of therapy (PT, OT, and ST combined) was 27.33 (SD = 6.52) h. All the patients received occupational therapy (OT) and speech therapy (ST) during their length of stay for 1 h per day per discipline. As demonstrated in Table 3, length of stay in days had a significant moderate positive correlation with PASS (r = 0.525, *p* = 0.04) and BBS scores (r = 0.546, *p* = 0.04). Length of stay was not correlated with 10MWT-SS (r = 0.04, *p* = 0.90) and 10WMT-F (r = 0.24, *p* = 0.39). Hours of therapy had a significant moderate positive correlation with PASS (r = 0.63, *p* = 0.01) and BBS (r = 0.63, *p* = 0.01) scores, weak correlation with the 10MWT-F (r = 0.32, *p* = 0.29), and no correlation with the 10MWT-SS (r = 0.12, *p* = 0.67).

## 4. Discussion

The results of this study showed that, in individuals with cerebellar infarct, treatment in inpatient rehabilitation had significant improvements in the PASS, BBS, 10MWT-SS, and 10MWT-F scores from admission to discharge when interventions focusing on gait training, balance training, neuromuscular re-education, and transfer training were implemented. Additionally, the average length of stay for individuals with a cerebellar stroke was 12.67 days. Length of stay demonstrated a moderate positive association with the PASS and BBS. 

Consistent with the previously published literature in patients with cerebellar stroke, inpatient rehabilitation improves balance and walking speed [17,18]. The results of this study are also corroborated by research demonstrating physical therapy interventions that include locomotion training are effective in decreasing fall risk and improving walking speed in patients with cerebellar stroke [4,6]. However, we add to the body of literature by demonstrating that, in addition to gait outcomes, inpatient rehabilitation improves balance assessed with the BBS and PASS. 

These results may assist clinicians in what interventions can be performed to improve balance, functional mobility, and gait speed for patients with cerebellar infarct in the inpatient rehabilitation setting. Current length of stay data may be helpful in guiding physical therapists to predict functional prognosis during initial evaluation of patients with cerebellar infarct in inpatient rehabilitation. The results of the current study will be more generalizable to this patient population as most of the published literature in rehabilitation of individuals with cerebellar infarct is case studies [4,17,18].

Additionally, the average length of stay for individuals with a cerebellar stroke was 12.67 days with an average of 27.33 h of combined physical, occupational and speech therapy. A moderate association between a longer length of stay was demonstrated with outcomes for the PASS and BBS. A study by Kelly et al. reported a 21.9-day stay for the same condition [5]. It should be noted that evidence suggests that there has been a trend of reduced length of stays for inpatient stroke rehabilitation patients from 45 to 28 days [6]. However, the results of a study by Joa and colleagues suggests that patients with cerebellar stroke display superior outcomes, such as shorter length of stay and greater functional status at admission and discharge, compared to other stroke subclassifications [19]. Collectively, the reduced length of stay compared to previously published studies may be due to changing trends in healthcare. 

Although there is no reported literature on the long-term effects of inpatient rehabilitation in patients with cerebellar infarct, the results of the study by Klassen et al. showed that higher walking doses during inpatient rehabilitation compared to usual care had lasting improvements with walking endurance 1 year after follow-up [20]. In patients with degenerative cerebellar disease, 4 weeks of inpatient rehabilitation has been shown to have lasting improvements in ataxia, gait speed, and walking ability at 12-week follow-up, with sustained improvements in normalized gait speed at 24-week follow-up [21].

The results of this study demonstrate that individuals who underwent inpatient rehabilitation for an acute cerebellar stroke demonstrated significant improvements in balance and gait. Interventions provided during rehabilitation included gait training consistent with high-intensity gait training and standing balance training. Additionally, average length of stay was 12.67 days, which was moderately associated with PASS and BBS outcomes. Given the limited literature in reporting the rehabilitation and outcomes of patients with acute cerebellar stroke, the results of this study have important clinical implications. However, limitations of the present study include the study being a retrospective chart review, the patient population being primarily older males, and a smaller sample size. Future studies may aim to examine the effect of specific interventions on gait and balance outcomes and complete this in a larger sample of individuals.

## Figures and Tables

**Table 1:** Interventions provided by each rehabilitation discipline.

Physical Therapy	
Gait Training	Goal of maximizing repetition and intensity via harness supported treadmill training and overground gait training—aim for 70–85% of HRMax
Balance Training	Static/dynamic balance training, including static standing on firm surface with eyes open or closed and static standing on compliant surface with eyes open and closed Stepping over obstacles without UE support Balance during dynamic stepping
Occupational Therapy	Activities of Daily Living Instrumental Activities of Daily Living UE reach and grasp standing vs. sitting UB ergometer
Speech-Language Pathology	Swallowing and feeding interventions Speech exercises Cognitive training

**Table 2:** Participant demographics.

	Male, n = 9 (%)	Female, n = 6 (%)	Total, n = 15 (%)
**Age (years)**	5 (33.3%)	1 (6.7%)	6 (40.0%)
60–69	4 (26.7%)	3 (20.0%)	7 (46.7%)
70–79	0 (0.0%)	2 (13.3%)	2 (13.3%)
80–89			
**Stroke Side**			
Left	3 (20.0%)	4 (26.7%)	7 (46.7%)
Right	2 (13.3%)	1 (6.7%)	3 (20.0%)
Unidentified	4 (26.7%)	1 (6.7%)	5 (33.3%)
**Anatomical Area**			
Cerebellum Only	8 (53.3%)	6 (40.0%)	12 (93.3%)
Cerebellum and Cerebrum	1 (6.7%)	0 (0.0%)	1 (6.7%)
**Ambulation Status on Admission**			
Household Ambulator	5 (33.3%)	5 (33.3%)	10 (66.7%)
Limited Community Ambulator	4 (26.7%)	1 (6.7%)	5 (33.3%)
Community Ambulator	0 (0.0%)	0 (0.0%)	0 (0.0%)
**Fall Risk on Admission**			
High	9 (60.0%)	6 (40.0%)	15 (100%)
Low	0 (0.0%)	0 (0.0%)	0 (0.0%)
**Ambulation Status on Discharge**			
Household Ambulator	1 (6.7%)	2 (13.3%)	3 (20.0%)
Limited Community Ambulator	5 (33.3%)	4 (26.7%)	9 (60.0%)
Community Ambulator	3 (20.0%)	0 (0.0%)	3 (20.0%)
**Fall Risk on Discharge**			
High	1 (6.7%)	4 (26.7%)	5 (33.3%)
Low	8 (53.3%)	2 (13.3%)	10 (66.7%)

**Table 3:** Pearson correlations.

	Age	PASS	BERG	10MWTSS	10MWTF	Days	Hours
Age	1	−0.064	0.109	−0.134	−0.367	−0.047	0.006
PASS	−0.064	1	0.845 **	0.468	0.595 *	0.525 *	0.633 *
BERG	0.109	0.845 **	1	0.349	0.580 *	0.546 *	0.634 *
10MWTSS	−0.134	0.468	0.349	1	0.499	0.035	0.119
10MWTF	−0.367	0.595 *	0.580 *	0.499	1	0.241	0.324
Days	−0.047	0.525 *	0.546 *	0.035	0.241	1	0.947 **
Hours	0.006	0.633 **	0.634 *	0.119	0.324	0.947 **	1

* significance *p* < 0.05, ** significance *p* < 0.01.

## Data Availability

Data are available upon request.

## Public Involvement Statement

No public involvement in any aspect of this research.

## Guidelines and Standards Statement

This manuscript was drafted against the CONSORT checklist.
